# Tissue Remodelling following Resection of Porcine Liver

**DOI:** 10.1155/2015/248920

**Published:** 2015-07-09

**Authors:** Ingvild Engdal Nygård, Kim Erlend Mortensen, Jakob Hedegaard, Lene Nagstrup Conley, Christian Bendixen, Baldur Sveinbjørnsson, Arthur Revhaug

**Affiliations:** ^1^Laboratory of Surgical Research, Institute of Clinical Medicine, University of Tromsø, 9037 Tromsø, Norway; ^2^Department of Digestive Surgery, University Hospital of North Norway, 9038 Tromsø, Norway; ^3^Department of Genetics and Biotechnology, Faculty of Agricultural Sciences, University of Aarhus, 8830 Tjele, Denmark; ^4^Department of Molecular Medicine, Aarhus University Hospital, Skejby, Palle Juul-Jensens Boulevard 99, 8200 Aarhus N, Denmark; ^5^Molecular Inflammation Research Group, Institute of Medical Biology, University of Tromsø, 9037 Tromsø, Norway

## Abstract

*Aim.* To study genes regulating the extracellular matrix (ECM) and investigate the tissue remodelling following liver resection in porcine. *Methods.* Four pigs with 60% partial hepatectomy- (PHx-) induced liver regeneration were studied over six weeks. Four pigs underwent sham surgery and another four pigs were used as controls of the normal liver growth. Liver biopsies were taken upon laparotomy, after three and six weeks. Gene expression profiles were obtained using porcine-specific oligonucleotide microarrays. Immunohistochemical staining was performed and a proliferative index was assessed. *Results.* More differentially expressed genes were associated with the regulation of ECM in the resection group compared to the sham and control groups. Secreted protein acidic and rich in cysteine (SPARC) and collagen 1, alpha 2 (COL1A2) were both upregulated in the early phase of liver regeneration, validated by immunopositive cells during the remodelling phase of liver regeneration. A broadened connective tissue was demonstrated by Masson's Trichrome staining, and an immunohistochemical staining against pan-Cytokeratin (pan-CK) demonstrated a distinct pattern of migrating cells, followed by proliferating cell nuclear antigen (PCNA) positive nuclei. *Conclusions.* The present study demonstrates both a distinct pattern of PCNA positive nuclei and a deposition of ECM proteins in the remodelling phase of liver regeneration.

## 1. Introduction

The liver is known for its unique capacity to regenerate, with multiple studies having been conducted to assess the genetic mechanisms controlling the early phases of liver regeneration, as well as the corresponding histological changes.

Significant changes in liver architecture during regeneration have been described such as the differential expression of cell-adhesion proteins, basement-membrane proteins, and changes in both intra- and intercellular junctions. As reviewed by Taub, the reformation of the normal liver architecture occurs only after restoration of the original liver mass [1]. Although the terminal phase of liver regeneration has just entered a phase of rapid discovery [2–11], few studies have focused on the reorganization of the liver architecture after a completed regenerative process.

Hepatocytes are known to be self-renewing under normal conditions, although the origin of hepatocytes under liver regeneration still remains controversial [12]. Hepatocyte proliferation is the first line of regenerative response to acute or early chronic liver injury [13, 14] and constitutes the fundamental driving force of liver regrowth [14]. Previous studies have suggested the “streaming liver hypothesis,” thus implying that new hepatocytes arise in the periportal area and then gradually migrate towards the pericentral area [12, 15]. Since this hypothesis remains controversial and has mainly been studied in rodents, we found it interesting to study the tissue remodelling after a PHx (partial hepatectomy) in a porcine model.

The primary aim of this study was to assess differentially expressed genes and histological changes after a completed regenerative process in porcine, by reinterpretation of an already established microarray analysis of gene expression profiles [2] focusing on genes associated with synthesis, formation, regulation, deposition, and degradation of the extracellular matrix, supplemented by an immunohistochemical assessment. Compared to rodents, the pig bears a closer genetic and physiological resemblance to man. In addition, the use of a chronic porcine model enables the researcher to study changes over time in the same individual, in contrast to rodent models, where several animals are usually sacrificed at various time points. This is especially important when studying gene expression as one research object (animal) may easily contaminate a common gene pool when performing comparative expression analysis over time. The secondary aim of this study was therefore to investigate if the origin of hepatocytes in porcine liver regeneration supports previous reports in rodent models.

## 2. Materials and Methods

### 2.1. Samples and Microarray Analysis

This study utilizes a previously established dataset of the transcriptional profiles in the terminal phase of porcine liver regeneration obtained using microarray technology. The experiment is described in detail in [2] and in brief in the following sections. In addition, an immunohistochemical analysis was performed to validate the transcriptional profiles.

### 2.2. Experimental Setup

Twelve female Norwegian landrace pigs, weighing 31.7 ± 5.13 kg (approximately 2 months of age) and from a single commercial farm, were used. As male piglets are surgically castrated shortly after birth and the technique may induce pain and long-lasting stress, we chose only female pigs in our study. The animals were housed in a closed-system indoor facility with a 55 ± 10% relative humidity, 17-18 air changes per hour, and a temperature of 20 ± 1°C. The pigs shared fence-line contact with another related pig and were singly housed in 1.5 × 1.5 m pens with* ad libitum* access to tap water from water nipples, liquid dietary supplements (milk replacement for piglets), and digestive energy mixed with water. Light was supplied on a 12:12-hour schedule.

The pigs were subject to a 60% PHx (Group 1), sham surgery (Group 2), and controls (Group 3, *n* = 4 each group).

This project was approved in agreement with the Norwegian Animal Welfare Act § 21 and The Norwegian Regulation on Animal Experimentation §§ 7, 8, and 13. Our department is run in agreement with the European Convention for the Protection of Vertebrate Animals used for Experimental and Other Scientific Purposes.

The procedures for the anaesthesia, perioperative monitoring, surgery, and recovery were performed as previously reported [2].

### 2.3. Biopsies

A reference sample was taken from all animals in all groups upon laparotomy, before PHx, and at time points three weeks after PHx and six weeks after PHx. Biopsies were both immediately immersed in RNAlater (Ambion) and preserved at −70°C until RNA extraction and microarray analysis and fixed in 4% paraformaldehyde for histological preparation.

### 2.4. Microarray Methods

The two-colour microarray experiment was conducted as a common reference design using a reference consisting of equal amounts of total-RNA from all samples. Alexa flour-labelled cDNA was synthesized from the 36 individual samples and from the reference followed by cohybridization to 27 K pig oligonucleotide microarrays representing approximately 20 K porcine genes. Following hybridization, scanning, and image analysis, log2-transformed ratios were imported into the R computing environment (version 2.6.1 for Windows) using the package Linear Models for Microarray Analysis (Limma, version 2.12.0, [16]) for normalization and statistical analysis to identify genes being significantly differentially expressed due to resection over time adjusting for effects by using the expression profiles obtained from the control animals and the sham operated animals. The set of genes was further analysed by retrieving genes associated with synthesis, formation, regulation, deposition, and degradation of the extracellular matrix at all time points.

A detailed description of the microarray experiment, together with the resulting dataset, is available at NCBI's Gene Expression Omnibus (GEO [17, 18], http://www.ncbi.nlm.nih.gov/geo) using the accession numbers GPL5972 (the used microarray) and GSE14396 (data).

### 2.5. Immunohistochemistry

Liver tissue sections were stained for *α*-SMA, Abcam (ab5694) (Cambridge, UK), SPARC, Cell Signaling Technology (D10F10) (Danvers, USA), COL-1A, Abcam (ab90395) (Cambridge, UK), pan-Cytokeratin, Ventana Medical Systems (AE1/AE3/PCK26) (Tucson, Arizona, USA) and PCNA, Cell Signaling Technology (PC10) (Danvers, USA).

Formalin-fixed and paraffin-embedded tissue sections were deparaffinized in xylene and graded alcohols, hydrated, and washed in phosphate buffered saline (PBS). After an antigen retrieval in a sodium citrate buffer (pH 6) in a microwave oven, the endogenous peroxidase was blocked by 0.3% H_2_O_2_ for 15 min. Sections were incubated overnight at 4°C with primary antibody (1 : 100) and as a secondary antibody; a horseradish peroxidase (HRP) SuperPicTure Polymer detection kit was used (Invitrogen). As a chromogen substrate for HRP, either DAB or ACE (Dako) was used. A matched isotype control was used as a control for nonspecific background staining, and a routine standard staining showing a normal histology of liver sections from respective groups was performed with hematoxylin/eosin and Masson's Trichrome, respectively. The proliferative index was assessed by counting the number of PCNA-positive cells at ×200 magnification in five randomly chosen fields per slide in three animals, one at 0, one at 3, and one at 6 weeks after PHx.

## 3. Results

### 3.1. Microarray Analysis

Overall, more differentially expressed genes were associated with the regulation of ECM in the resection group compared to the sham and control groups. All genes regulating ECM are presented in Table 1.

When comparing gene expression at the time contrast 3–0 weeks in between groups, we detected that the number of genes regulating ECM in the resection group differed from the sham and control groups (Table 1). At time contrast 3–0 weeks, six differentially expressed genes were regulating ECM in the resection group, whereas only one gene was regulating ECM in the sham and the control group.

#### 3.1.1. Regulation of ECM Genes in the Resection Group

In our study we have focused on genes with a log fold-change (log FC) over 0.7 and genes repeatedly expressed in the beginning of the experiment, whereas all genes at the end of the experiment are discussed.

Of the six genes differentially expressed at the time contrast of 3–0 weeks, four were solely expressed in the resection group, and one of these genes was repeatedly expressed at the time contrast of 6–3 weeks. The gene repeatedly expressed was SPARC (secreted protein acidic and rich in cysteine, osteonectin), which is a matrix-associated protein that elicits changes in cell shape, inhibits cell-cycle progression, and influences the synthesis of ECM [20, 23]. This gene was upregulated during the three first weeks after PHx (log FC 0.78) and downregulated at the remodelling phase of regeneration (log FC of −0.7).

Another gene solely expressed in the resection group was COL1A2 (collagen, type I, alpha 2). COL1A2 acts as a structural component of the ECM and together with COL1A1 and COL1A2 encodes the fibrillar collagen type I, while accounting for 36% of the total collagens in the ECM of healthy liver [19]. COL1A2 was upregulated during the three first weeks after PHx (log FC 0.84) but not differentially expressed at the remodelling phase of regeneration.

#### 3.1.2. Regulation of ECM Genes in the Sham Group

Four genes were differentially expressed solely in the sham group. By comparing the first time contrast of 3–0 weeks with the second of 6–0 weeks, we found one common upregulated gene, BMP1 (bone morphogenetic protein 1). Moreover, BMP1 is involved in the formation of the extracellular matrix [25].

At the time contrast of 6–3 weeks, two genes were downregulated, DSP (desmoplakin) and ITGAV (integrin, alpha V).

Desmoplakin is a cell surface adhesion protein. During the regeneration of rat hepatocytes, desmosomes between neighbouring cells remain constant [28].

ITGAV is a member of the integrin family, and the integrin-signaling pathway participates in regulating hepatocyte proliferation during rat liver regeneration [31]. The gene is also reported to interact with receptors in the ECM [23]. STEAP1 (six-transmembrane epithelial antigen of the prostate 1) was upregulated towards the end of the experiment.

#### 3.1.3. Regulation of ECM Genes in the Control Group

Four genes were differentially expressed solely in the control group. By comparing the first with the second time contrast, we found one commonly upregulated gene, STEAP1. This gene was also upregulated in the sham group at time contrast 6–3 weeks. The gene product is predicted to be a six-transmembrane protein and shown to be a cell surface antigen significantly expressed at cell-cell junctions [27].

Towards the end of the experiment, one gene was downregulated, MMP2 (matrix metalloproteinase 2). Proteins of the matrix metalloproteinase (MMP) family are involved in the breakdown of extracellular matrix in liver repair reactions [30].

### 3.2. Immunohistochemical Analysis

#### 3.2.1. Nontreated Controls

**Figure 1:**
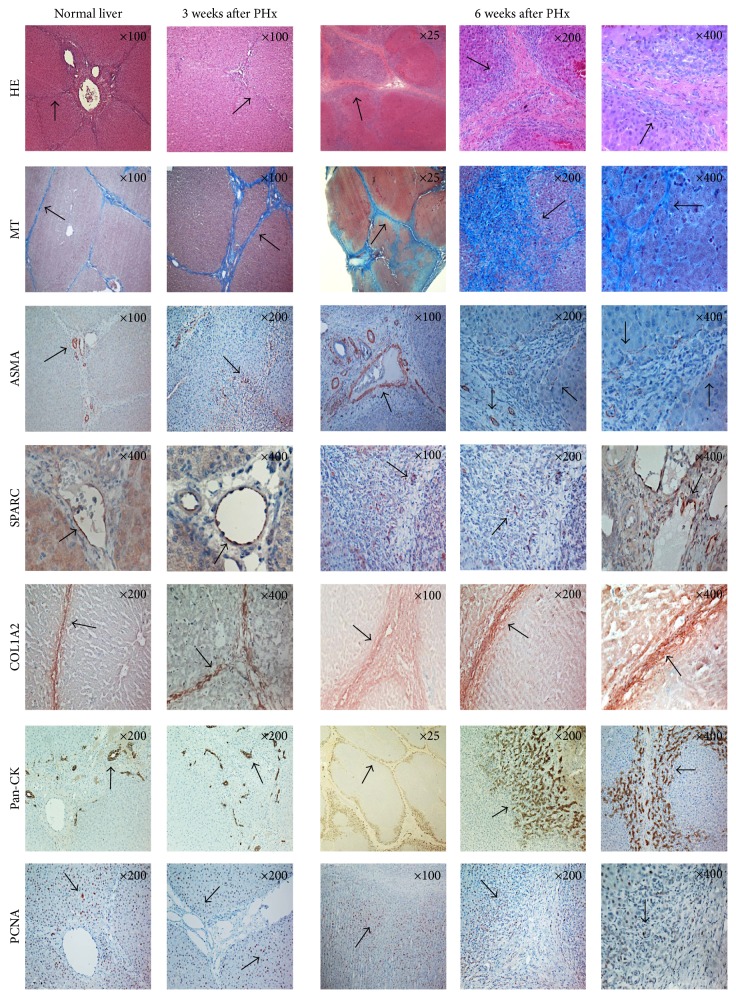
Overview of immunohistochemically stained normal and regenerated liver, three and six weeks after PHx. Staining for HE, Masson's Trichrome, *α*-SMA, SPARC, COL1A2, pan-CK and PCNA of a normal liver, a regenerated liver three weeks after PHx, and a regenerated liver six weeks after PHx, respectively. Original magnification at the upper right corner.

As seen in Figure 1, HE staining demonstrated the hepatic parenchyma being divided into lobules consisting of a hexagonal arrangement of hepatocytes. At the verticals of the lobules, there were portal triads containing a bile duct, hepatic artery, and portal vein. The hexagons were divided by a fine, delicate lining of connective tissue. By Masson's Trichrome staining, the connective tissue was revealed in blue. *α*-SMA (alpha smooth muscle actin), which is expressed in activated hepatic stellate cells and smooth muscle cells, was positive in a linear pattern both along the connective tissue and within the vascular walls. Pan-CK staining revealed positive bile duct cells located within the connective tissue. The matrix-associated protein SPARC was positively stained in endothelial cells of vascular walls within the connective tissue, and COL1A2 revealed heavily positive cells all over the connective tissue. PCNA-positive cells exhibited a scattered distribution throughout the liver tissue during normal liver growth (Figure 1).

#### 3.2.2. Three Weeks after PHx

**Figure 2:**
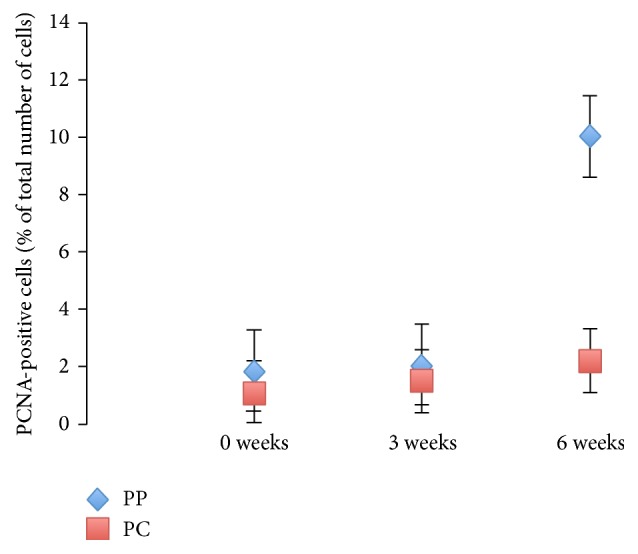
PCNA index. Percentage of PCNA-positive cells located in the periportal region (PP) and pericentral region (PC), at 0, 3, and 6 weeks after PHx.

HE staining three weeks after PHx demonstrated a presence of hepatocyte-like cells in the connective tissue septa, hence making the hexagonal lobules less visible, whereas Masson's Trichrome revealed that the connective tissue septa were broadened. In addition to the linear staining of *α*-SMA along the connective tissue, there were also *α*-SMA-positive cells in between the hepatocytes. As in a normal liver, the pan-CK staining identified positive bile duct cells located within the connective tissue but differed from normal liver due to the noncircular shape and additional number of bile ducts. As in a normal liver, SPARC-positive cells were identified in the endothelial cells of vascular walls within the connective tissue. COL1A2 staining was positive all over the broadened connective tissue, though PCNA-positive nuclei differed in distribution throughout the parenchyma, as most positive nuclei were aligned in the parenchyma along the broadened connective tissue in the periportal region (Figures 1 and 2).

#### 3.2.3. Six Weeks after PHx

**Figure 3:**
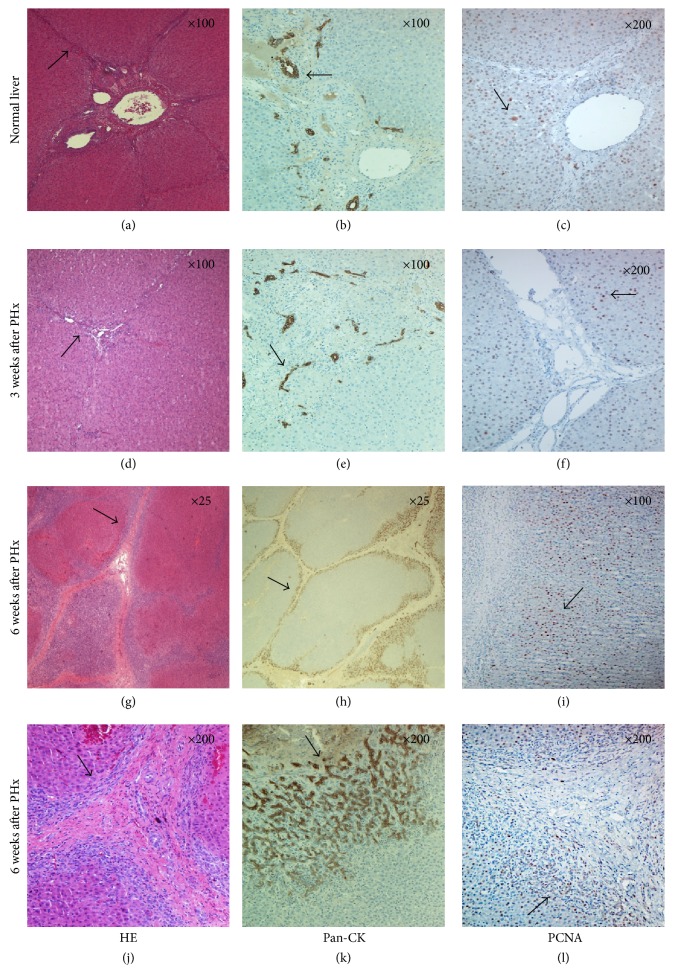
HE, pan-CK, and PCNA staining throughout liver regeneration. (a, d, g, and j) HE staining of normal liver and regenerated liver. Note the broadening of the connective tissue at six weeks after PHx. (b, e, h and k) Pan-CK staining of normal and regenerated liver. Note the heavily stained cholangiocytes in the periportal region at six weeks after PHx. (c, f, i, and l) PCNA staining of normal and regenerated liver. Note the multiple cells immunopositive for PCNA presented along the connective tissue at six weeks after PHx. Original magnification at the upper right corner.

HE staining at six weeks after PHx demonstrated islands of hepatocyte-like cells in between the broadened connective tissue, thus making the structural units of liver lobules less organized. Along the connective tissue, multiple PCNA-positive cells were presented. Masson's Trichrome staining revealed a broadened and reestablished connective tissue capsule, dividing the parenchyma into separated nodular compartments (Figure 1). This pattern was repeatedly demonstrated with pan-CK staining, revealing a distinct pattern of hepatic compartmentalization. At an original magnification ×200, the pan-CK staining differed, from heavily stained cholangiocytes in the periportal region towards lightly stained cholangiocytes in the pericentral region (Figures 3(h) and 3(k)).

Like three weeks after PHx, *α*-SMA-positive cells were identified both in the connective tissue and in between the hepatocytes. SPARC-positive cells were located all over the unorganized and broadened connective tissue, and COL1A2 staining was also positive in the broadened connective tissue (Figure 1).

PCNA revealed a pattern of multiple heavily stained nuclei along the connective tissue compared with the staining of nuclei in the pericentral region (Figures 2, 3(i), and 3(l)).

**Figure 4:**
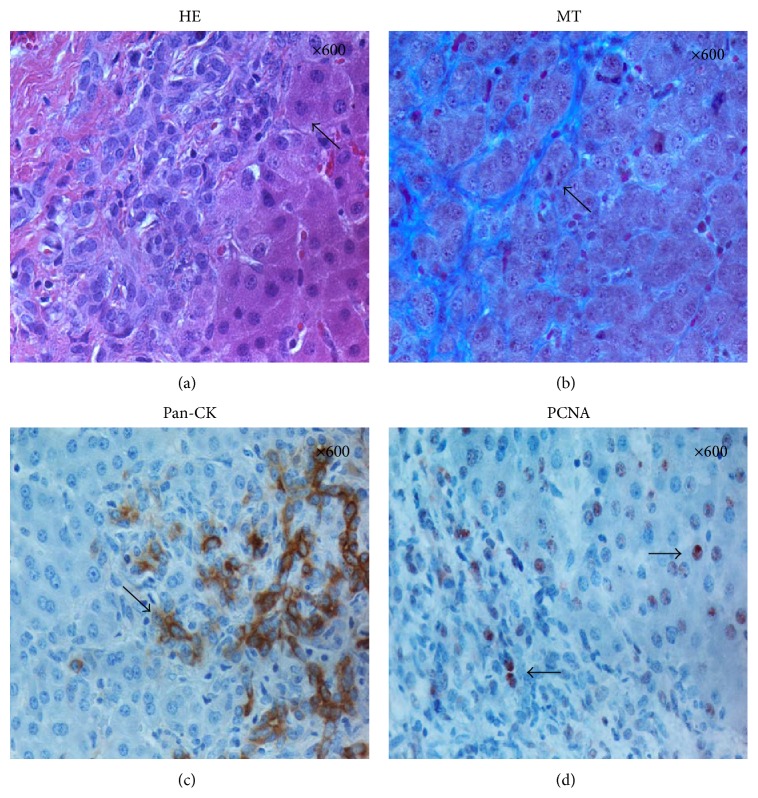
HE, MT, pan-CK, and PCNA at six weeks after PHx. (a and b) HE and Masson's Trichrome staining revealed hepatocyte-like cells located in between the fibres of the connective tissue. (c and d) Pan-CK and PCNA-positive cells located both in the connective tissue layer and in the adjacent hepatocyte parenchymal tissue. Original magnification at the upper right corner.

At high magnification, hepatocyte-like cells were observed in between the fibers of the connective tissue (Figures 4(a) and 4(b)). In the same area, a significant number of cells immunopositive for pan-Cytokeratin and PCNA were detected in the connective tissue layer and also in the adjacent hepatocyte parenchymal tissue (Figures 4(c) and 4(d)).

## 4. Discussion

Since the terminating phase of liver regeneration is now entering a phase of rapid discovery [2–11], our aim was to study differentially expressed genes regulating the extracellular matrix, together with the tissue remodelling of normal liver architecture following a completed regeneration process in porcine.

During the first three weeks after PHx, microarray analysis revealed that SPARC and COL1A2 were differentially expressed with a log FC of 0.78 and 0.84, respectively. The matrix-associated protein SPARC is known to influence the synthesis of the extracellular matrix, which is one of the main components of the connective tissue [20, 32]. Taken together with the immunopositive SPARC cells within the connective tissue, the early genetic upregulation of SPARC confirms the function of SPARC in extracellular matrix formation. Staining with Masson's Trichrome corroborates these results with a broadening of the connective tissue septa at three weeks after PHx (Figure 1).

COL1A2 acts as a structural component of the ECM, with the collagen family of ECM proteins playing a vital role in maintaining the structure of the liver [19]. Interestingly, COL1A2 was upregulated during the three first weeks after PHx, followed by a demonstration of COL1A2-positive cells within the connective tissue three weeks after PHx (Table 1, Figure 1). The presence of COL1A2-positive cells and the high differential expression of COL1A2 suggest that collagen production may be important during liver regeneration. Myofibroblasts are suggested to derive from HSC (hepatic stellate cells) located in the perisinusoidal space, and after a liver injury the HSC are activated and the presence of myofibroblasts is reported to be prominent [33]. According to Hernandez-Gea and Friedman, HSC are a primary source of ECM after liver injury [34]. The fact that *α*-SMA, a marker of HSC and smooth muscle cells, revealed positive cells along the connective tissue, in addition to *α*-SMA-positive cells in between the hepatocytes, not only implicates the presence of activated HSC, but could also explain the broadening of the connective tissue as part of a liver injury-induced production of ECM.

During the remodelling phase of liver regeneration, the matrix-associated gene SPARC was negatively expressed with a log FC of −0.7. According to Steer, the extracellular matrix plays an important role in maintaining growth arrest in the adult liver, as well as regulating liver regeneration. The synthesis and deposition of the different matrix components are part of restoring the normal microarchitecture of the liver [35]. In addition, SPARC is required for proper ILK (integrin linked kinase) activation [36]. Donthamsetty et al. reported that the lack of ILK in mice hepatocytes was followed by a prolonged proliferative response related to liver regeneration [6]. The downregulation of SPARC during the remodelling phase of regeneration as seen in our study may involve a lack of ILK activation; hence the proliferative response may continue for more than six weeks. Stolz et al. conducted a study of liver regeneration after a 70% partial PHx in rats [37] and reported that SPARC was known to be synthesized by endothelial and stellate cells within the space of Disse, in addition to being implicated in the regulation of angiogenesis and wound healing. They demonstrated that SPARC decreased at the SEC (sinusoidal endothelial cell) membrane 72 hours after PHx when compared to nonresected livers but was not seen in any other fractions. These findings corroborate our present results in a porcine model, with a late downregulation of SPARC in the remodelling phase of regeneration. Our observations in porcine, along with previous reports in rodents, demonstrate that the ECM plays an important role in the remodelling of liver regeneration in all species.

Validation by immunohistochemical staining revealed SPARC-positive cells within the connective tissue at six weeks after PHx (Figure 1). Masson's Trichrome staining revealed a broadened connective tissue at six weeks after PHx (Figure 1), which may be a result of the early upregulation of SPARC in the initial step of liver regeneration. The fact that SPARC is upregulated in the initial phase of regeneration, leaving a broadened connective tissue at three and six weeks after PHx, indicates a role in the early deposition of SPARC-associated matrix proteins, thereby contributing in growth arrest of the regenerating liver.

The genetic expression of COL1A2 was absent at three and six weeks after PHx, indicating a role in the prevention of a continued regeneration process. The immunohistochemical staining of COL1A2 demonstrated heavily positive collagen cells within the broadened connective tissue at six weeks after PHx (Figure 1), which may indicate that collagens play a role in growth arrest in the remodelling phase of liver regeneration.

As seen three weeks after PHx, *α*-SMA-positive cells were identified in both the connective tissue and between the hepatocytes (Figure 1), hence indicating the presence of activated HSC, also after a completed regenerative process.

Interestingly, several other genes regulating the ECM were differentially expressed in the sham and control groups. This in turn is tentatively an indication of the fact that the normal growing, nonresected liver is under constant control by the opposing actions of genes regulating synthesis, formation, regulation, deposition, and degradation of the extracellular matrix as required.

According to Furuyama et al., cholangiocyte-like Sox9-positive cells could function as hepatocyte progenitors after injury [38]. They suggest that hepatocytes were derived from Sox9-positive cells that were cholangiocytes in their initial morphology. In our study, we demonstrate the presence of pan-CK-positive cells six weeks after injury (PHx) to be in a migrating pattern from heavily stained cells in the portal region, fading towards lightly stained cells in the central area (Figure 4(c)). The “streaming liver” hypothesis suggests that new hepatocytes arise in the periportal area and then gradually migrate towards the pericentral area [12]. In addition to a broadening of the connective tissue at six weeks after PHx, our study exhibits multiple PCNA-positive nuclei along the parallel periportal parenchyma. As reported by Cardinale et al., hepatic stem cells are reported to localize in the canals of Hering, which differentiate into hepatic progenitor cells for both cholangiocytes and hepatocytes [39]. Hepatocyte progenitor cells and normal biliary epithelial cells are strongly immunopositive for cytokeratins [40]. Taken together with the PCNA-positive nuclei localized in the same area, our results, which lean heavily towards lightly stained pan-CK-positive cells within the parenchyma being distributed in parallel to the connective tissue, suggest the periportal parenchyma to be the zone of proliferation during remodelling of the liver. Some previous reports have described this pattern as “the streaming hypothesis,” and our observations are in line with these reports.

Over the past decade, microarray analysis has gained acceptance as a standard tool for studies in molecular biology. However, the use of microarray and fold-change has the disadvantage that it needs cut-off values. Therefore it does not include all biologically present genes, as some might have a very small fold-change. By contrast, it is unlikely that very small fold-changes have any biological relevance. With regard to the study outline, the three-week periods in between biopsy sampling raise the uncertainty of whether other genes have been differentially expressed in between sampling. Even so, despite the lack of the expression of these genes in our study, we cannot exclude the presence or biological relevance of previously reported genes regulating the ECM in the remodelling phase of liver regeneration.

## 5. Conclusions

Our study in porcine, along with previous reports in rodents, demonstrates that the ECM plays an important role in the remodelling phase of liver regeneration in all species. In addition, our data support previous reports in rodent models, as they are in line with “the streaming hypothesis” that suggests a pattern of hepatocyte migration from the periportal to the pericentral zone of the liver lobule.

## Figures and Tables

**Table 1:** Differentially expressed genes regulating ECM in all groups over time.

Resection group	Upregulated (log FC)	Downregulated (log FC)	Function	Reference
3–0 weeks	COL1A2 (0.84)		Acts as a structural component of the ECM	[19]
	SPARC (0.78)		Influences the synthesis of ECM	[20]
	TCF4 (0.33)		Is highly expressed in connective tissue fibroblasts	[21]
	ERBB3 (0.26)		Involved in regulating the response of fibroblasts	[22]
	TFPI2 (0.26)		Is an ECM structural constituent	[23]
	TIMP (0.14)		Play a key role in maintaining the balance between ECM deposition and degradation	[24]

6–3 weeks		SPARC (−0.7)	Influences the synthesis of ECM	[20]

Sham group				

3–0 weeks	BMP1 (0.22)		Involved in ECM formation	[25]

6–0 weeks	GNG11 (0.35)		Induces cellular senescence in normal human fibroblasts	[26]
	BMP1 (0.23)		Involved in ECM formation	[25]

6–3 weeks	STEAP1 (0.29)		A cell surface antigen expressed at cell-cell junctions	[27]
		ITGAV (−0.15)	Interacts with receptors in the ECM	[23]
		DSP (−0.47)	Desmoplakin is a cell surface adhesion protein	[28]

Control group				

3–0 weeks	STEAP 1 (0.48)		A cell surface antigen expressed at cell-cell junctions	[27]

6–0 weeks	STEAP1 (0.65)		A cell surface antigen expressed at cell-cell junctions	[27]
	F11R (0.54)		Encodes JAM-A, a transmembrane protein interacting with molecules in the ECM	[29]
	OCLN (0.4)		Encodes occludin, a transmembrane protein interacting with molecules in the ECM	[29]
	TFPI2 (0.38)		Is an ECM structural constituent	[23]
	TCF4 (0.28)		Is highly expressed in connective tissue fibroblasts	[21]

6–3 weeks		MMP2 (−0.44)	Involved in the breakdown of ECM in liver repair reactions	[30]
